# Efficacy and safety of lumasiran for infants and young children with primary hyperoxaluria type 1: 30-month analysis of the phase 3 ILLUMINATE-B trial

**DOI:** 10.3389/fped.2024.1392644

**Published:** 2024-09-16

**Authors:** Yaacov Frishberg, Wesley Hayes, Hadas Shasha-Lavsky, David J. Sas, Mini Michael, Anne-Laure Sellier-Leclerc, Julien Hogan, Richard Willey, John M. Gansner, Daniella Magen

**Affiliations:** ^1^ Division of Pediatric Nephrology, Shaare Zedek Medical Center and Faculty of Medicine, Hebrew University of Jerusalem, Jerusalem, Israel; ^2^ Department of Paediatric Nephrology, Great Ormond Street Hospital, London, United Kingdom; ^3^ Pediatric Nephrology Unit, Galilee Medical Center, Nahariya, Israel; ^4^ Azrieli Faculty of Medicine, Bar Ilan University, Safed, Israel; ^5^ Division of Pediatric Nephrology and Hypertension, Mayo Clinic, Rochester, MN, United States; ^6^ Division of Pediatric Nephrology, Baylor College of Medicine, Texas Children’s Hospital, Houston, TX, United States; ^7^ Hôpital Femme Mère Enfant and Centre d’Investigation Clinique Inserm, Hospices Civils de Lyon, ERKnet, Bron, France; ^8^ Pediatric Nephrology Department, Hôpital Robert-Debré, APHP, Paris, France; ^9^ Biostatistics, Alnylam Pharmaceuticals, Cambridge, MA, United States; ^10^ Clinical Development, Alnylam Pharmaceuticals, Cambridge, MA, United States; ^11^ Pediatric Nephrology Institute, Rambam Health Care Campus, and Faculty of Medicine, Technion – Israel Institute of Technology, Haifa, Israel

**Keywords:** kidney, liver, lumasiran, oxalate, pediatric, rare diseases, RNA interference, primary hyperoxaluria type 1

## Abstract

**Background:**

Primary hyperoxaluria type 1 (PH1) is a genetic disorder resulting in overproduction of hepatic oxalate, potentially leading to recurrent kidney stones, nephrocalcinosis, chronic kidney disease, and kidney failure. Lumasiran, the first RNA interference therapeutic approved for infants and young children, is a liver-directed treatment that reduces hepatic oxalate production. Lumasiran demonstrated sustained efficacy with an acceptable safety profile over 12 months in infants and young children (age <6 years) with PH1 in ILLUMINATE-B (clinicaltrials.gov: NCT03905694), an ongoing, Phase 3, multinational, open-label, single-arm study.

**Methods:**

Here, we report interim efficacy and safety findings from ILLUMINATE-B following 30 months of lumasiran treatment. Eligible patients had an estimated glomerular filtration rate (eGFR) >45 ml/min/1.73 m^2^ if ≥12 months old or normal serum creatinine if <12 months old, and a urinary oxalate to creatinine ratio (UOx:Cr) greater than the upper limit of normal. All 18 patients enrolled in ILLUMINATE-B completed the 6-month primary analysis period, entered an extension period of up to 54 months, and continue to participate in the study.

**Results:**

At Month 30, mean percent change from baseline in spot UOx:Cr was −76%, and mean percent change in plasma oxalate was −42%. eGFR remained stable through Month 30. In 14 patients (86%) with nephrocalcinosis at baseline, nephrocalcinosis grade improved at Month 24 in 12; no patient worsened. In the 4 patients without baseline nephrocalcinosis, nephrocalcinosis was absent at Month 24. Kidney stone event rates were ≤0.25 per person-year through Month 30. Mild, transient injection site reactions were the most common lumasiran-related adverse events (17% of patients).

**Conclusion:**

In infants and young children with PH1, long-term lumasiran treatment resulted in sustained reductions in urinary and plasma oxalate that were sustained for 30 months, with an acceptable safety profile. Kidney function remained stable, low kidney stone event rates were observed through Month 30, and nephrocalcinosis grade improvements were observed through Month 24.

**Clinical Trial Registration:**

https://clinicaltrials.gov, identifier NCT03905694.

## 1 Introduction

Primary hyperoxaluria type 1 (PH1; OMIM #259900) is an autosomal recessive disease resulting from excess production of hepatic oxalate, potentially leading to kidney stones, nephrocalcinosis, and eventually chronic kidney disease, kidney failure, and deposition of calcium oxalate crystals in body organs, including bone, heart, and eyes (systemic oxalosis) (1–5). The phenotype is variable, with high mortality associated with infantile oxalosis (4, 6–8). Symptoms of PH1, as well as hyperhydration treatment, are associated with a substantial burden, negatively impacting quality of life (9, 10). Prompt diagnosis and treatment are critical to reduce oxalate production and mitigate the impact of excess oxalate on the kidneys and other organs (8).

Historically, treatment for PH1 has consisted mainly of supportive measures to delay or minimize oxalosis, and reactive measures to address ongoing oxalosis and associated damage (2, 11). Patients not on dialysis may be treated with hyperhydration and crystallization inhibitors, and pyridoxine (vitamin B6) may be administered. However, pyridoxine may only be effective in pyridoxine-responsive patients (e.g., those with the c.508 G>A [p.Gly170Arg] mutation (4, 12). Hemodialysis to reduce oxalate levels in the blood becomes essential as kidney function deteriorates, but it is often insufficient to prevent manifestations of systemic oxalosis (1, 13, 14). Replacement of the defective native liver carries significant risk of morbidity and mortality (1, 15).

Lumasiran, an RNA interference (RNAi) therapeutic (ie, one involving targeted inhibition of gene expression) that is directed to the liver (16), has been approved in the European Union “for the treatment of PH1 in all age groups” (17) and in the United States “for the treatment of PH1 to lower urinary oxalate (UOx) and plasma oxalate (POx) in pediatric and adult patients” (18). Lumasiran consists of a double-stranded small interfering RNA that is covalently linked to triantennary N-acetylgalactosamine (GalNAc), allowing for targeted delivery to the liver (16, 19, 20). In PH1, glyoxylate levels are increased due to pathogenic variants in the *AGXT* gene and deficient activity of AGT, an enzyme that metabolizes glyoxylate to glycine (21). Lumasiran causes the mRNA-encoding glycolate oxidase (GO; OMIM #605023) to be degraded, hence reducing glyoxylate, a substrate for oxalate production (19).

The lumasiran clinical development program in PH1 comprises 5 clinical trials in which a total of 98 patients were enrolled, including people of different ages and degrees of PH1 severity (19). The Phase 3, single-arm ILLUMINATE-B study (NCT03905694) is being conducted to examine lumasiran's efficacy and safety in infants and young children (age <6 years) with PH1 and estimated glomerular filtration rate (eGFR) >45 ml/min/1.73 m^2^ (22, 23). During the 6-month primary analysis period, lumasiran demonstrated clinically important reduction relative to baseline in spot urinary oxalate to creatinine ratio (UOx:Cr) by 72% (22). The most common treatment-related adverse events (AEs) were transient, mild injection site reactions (22). After 6 more months of treatment, during a long-term extension period, the efficacy and safety of lumasiran were maintained (23).

Here, we report efficacy and safety findings from ILLUMINATE-B following 30 months of lumasiran treatment.

## 2 Materials and methods

### 2.1 Study design and patients

ILLUMINATE-B is an ongoing, Phase 3, multinational, open-label, single-arm study. A primary analysis was conducted at 6 months; patients are now in an extension period of up to 54 months. The study design and eligibility criteria have been described previously (22, 23). Briefly, eligible patients had a genetically confirmed diagnosis of PH1, were <6 years old at study entry, had an eGFR >45 ml/min/1.73 m^2^ if ≥12 months old or normal serum creatinine if <12 months old, and a UOx:Cr greater than the upper limit of normal (ULN) for age. Lumasiran was administered subcutaneously according to a dosing regimen based on body weight (Table 1). All patients received lumasiran as 3 loading doses, once monthly (at Day 1, at Month 1, and at Month 2) at a dose based on body weight category, then received lumasiran either once monthly (patients weighing <10 kg) or once every 3 months (patients weighing ≥10 kg) at the maintenance dose, beginning at Month 3.

**Table 1:** Dosing regimen of lumasiran.

Body weight	Loading dose	Maintenance dose (begin 1 month after the last loading dose)
<10 kg	6.0 mg/kg once monthly for 3 doses	3.0 mg/kg once monthly
10 kg to <20 kg	6.0 mg/kg once monthly for 3 doses	6.0 mg/kg once every 3 months (quarterly)
≥20 kg	3.0 mg/kg once monthly for 3 doses	3.0 mg/kg once every 3 months (quarterly)

### 2.2 Details of ethics approval

The study protocol and amendments and informed consent form were reviewed and approved by Independent Ethics Committees/Institutional Review Boards prior to commencement of the study. This study was conducted in accordance with Good Clinical Practice as defined by the International Council on Harmonisation, the principles defined in the Declaration of Helsinki and its amendments, and all applicable national and international laws. Legal guardians provided informed consent and patients provided assent per local regulations and institutional standards.

### 2.3 Endpoints

The primary endpoint was percent change in spot UOx:Cr from baseline to Month 6, as described previously (22). Spot urine samples were used as an alternative to 24-hour UOx levels due to the inability of young children to comply with 24-hour urine collections (24–26). Secondary endpoints assessed in the extension period included absolute and percent change from baseline in UOx excretion, proportion of patients with UOx excretion less than or equal to the ULN and ≤1.5 × ULN for age, absolute and percent change from baseline in POx, and change from baseline in eGFR (23). The ULN for spot UOx:Cr is age-dependent and was based on Matos et al. (1999) (27) to account for an age-related decline that occurs in infants and young children. Exploratory endpoints included changes in nephrocalcinosis grade, kidney stone event rates, and plasma glycolate (23).

### 2.4 Assessments

Spot UOx and plasma glycolate were measured with validated liquid chromatography-tandem mass spectrometry (LC-MS/MS) assays. Spot UOx was expressed relative to creatinine (spot UOx:Cr). POx was measured with a novel, validated LC-MS/MS assay (28). eGFR was calculated for patients ≥12 months old using the Schwartz Bedside formula (29); eGFR was not calculated for patients <12 months old as the Schwartz Bedside formula is not validated for that age group (29). Drug antibodies against lumasiran were evaluated in plasma using a validated enzyme-linked immunoassay.

Kidney stone events were adjudicated by the investigator. A kidney stone event was defined as an event that included ≥1 of the following: visit to healthcare provider because of a kidney stone, medication for renal colic, stone passage, or macroscopic hematuria due to a kidney stone.

Renal ultrasounds were performed at baseline and Months 6, 12, and 24 (but not Month 30) and read by a central radiologist. Changes from baseline in nephrocalcinosis grade were categorized as follows, accounting for both kidneys: no change (stable), improving (which was further categorized into improving and improving to complete resolution), worsening, and indeterminate (defined as 1 kidney improving and 1 worsening).

### 2.5 Statistical analysis

This analysis was conducted using data as of a cutoff date of April 29, 2022, after all active study patients had completed their Month 30 visit.

All efficacy analyses were conducted in the efficacy analysis set, defined as all patients who received any amount of lumasiran and had ≥1 valid spot UOx:Cr value at baseline and ≥1 valid spot UOx:Cr value from assessments at Month 3 to Month 6. Percent and absolute change in POx from baseline were additionally analyzed in the POx analysis set, which included only patients in the efficacy analysis set whose baseline POx was ≥1.5 times the lower limit of quantitation (LLOQ; 5.55 µmol/L). The kidney stone event rate was calculated as the total number of kidney stone events divided by the total patient exposure time (events per person-year). The 95% CI for the kidney stone event rate was obtained using a generalized linear model for a Poisson distribution unless the rate was 0, in which case the upper bound of the 95% CI was calculated using the exact Poisson method.

A pyridoxine-responsive (PR) genotype was defined as NM_000030.3(AGXT):c.508G>A (p.Gly170Arg) or NM_000030.3(AGXT):c.454T>A (p.Phe152Ile), where N denotes nonsense and M denotes missense (30).

Cumulative safety data from the first dose of lumasiran through the data cutoff date are reported. Safety analyses were conducted in the safety analysis set, defined as all patients who received any amount of lumasiran. Duration of exposure to study drug was calculated using calendar months [duration of treatment (days)/30.44], whereas for study visits, 1 month was defined as 4 weeks (28 days).

All statistical analyses were performed using validated SAS statistical software, version 9.4.

## 3 Results

### 3.1 Patients

All 18 patients who enrolled in the study entered the extension and continue to participate. Baseline demographic and clinical characteristics are shown in Table 2.

**Table 2:** Baseline demographic and clinical characteristics.

Characteristic	All treated (*N* = 18)
Age at consent, median (range), months	50.1 (3–72)
Age at diagnosis, median, months	16.3
Time from diagnosis to first dose date, median, months	23.5
Genotype,^a^ *n* (%)	
PR/*	3 (17)
M/M or M/N	10 (56)
N/N	5 (28)
Pyridoxine use, *n* (%)	11 (61)
Spot UOx:Cr, median (range), mmol/mmol^b,c^	0.469 (0.166–1.708)
24-hour UOx corrected for BSA, mean (SEM), mmol/24 h/1.73 m^2^	2.083 (0.3170)
POx, median (range), µmol/L^d^	11.5 (6.6–30.6)
eGFR, median (range), ml/min/1.73 m^2^^e^	111 (65–174)
History of kidney stone events in past 12 months, *n* (%)	3 (17)
Presence of nephrocalcinosis at baseline, *n* (%)	14 (78)

^a^ PR was defined as NM_000030.3(AGXT):c.508G>A (p.Gly170Arg) or NM_000030.3(AGXT):c.454T>A (p.Phe152Ile). M and N were defined based on a publication by Mandrile et al. (30). The asterisk (*) denotes any genotype of PR, M, or N. M, missense; N, nonsense; PR, pyridoxine-responsive.

^b^ 1 mmol/mmol = 0.796 mg/mg.

^c^ Age-related reference ranges in spot UOx:Cr: <1 year, 0.015–0.26 mmol/mmol; 1 to <5 years, 0.011–0.12 mmol/mmol; 5 to 12 years, 0.06–0.15 mmol/mmol (11, 31).

^d^ ULN = 12.11 μmol/L for POx, as determined based on data from 75 healthy adults (22).

^e^ eGFR was calculated based on the Schwartz Bedside formula (29) for patients ≥12 months, *N* = 16; eGFR was not calculated for 2 patients because their age at baseline was <12 months.

BSA, body surface area; eGFR, estimated glomerular filtration rate; POx, plasma oxalate; SEM, standard error of the mean; UOx:Cr, urinary oxalate:creatinine ratio.

### 3.2 Efficacy

**Figure 1:**
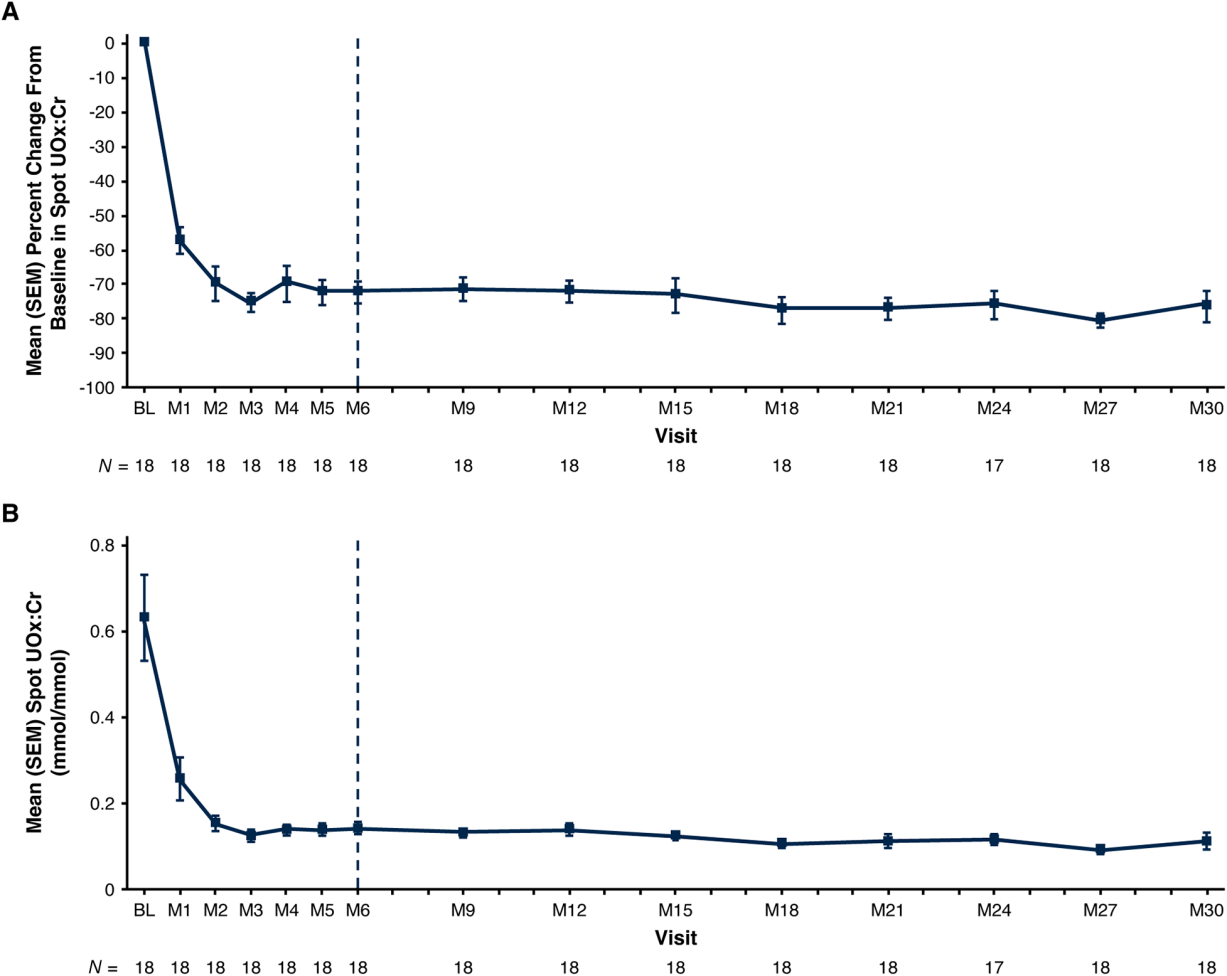
Mean (SEM) spot UOx:Cr. **(A)** Percent change from baseline at each visit and **(B)** actual values at each visit.^a^ Baseline value represents the mean of all assessments collected prior to the first dose of lumasiran; 1 mmol/mmol = 0.796 mg/mg; 1 mmol/mmol = 1,000 mmol/mol. End of the primary analysis period is represented by the vertical dashed line. ^a^The ULN for spot UOx:Cr is age-dependent (27). Age-related reference ranges in spot UOx:Cr: < 1 year, 0.015–0.26 mmol/mmol; 1 to <5 years, 0.011–0.12 mmol/mmol; 5 to 12 years, 0.06–0.15 mmol/mmol (11, 31). BL, baseline; M, month; SEM, standard error of the mean; ULN, upper limit of normal; UOx:Cr, urinary oxalate:creatinine ratio.

Mean spot UOx:Cr decreased from 0.63 mmol/mmol at baseline to 0.11 mmol/mmol at Month 30; mean [standard error of the mean (SEM)] percent change from baseline was −75.8% (4.5%) (Figure 1; Table 3). Thirteen of 18 patients (72%) had spot UOx:Cr values ≤1.5 × ULN at Month 30, and 7 (39%) had spot UOx:Cr values ≤ ULN (Table 3). The percent change from baseline in 24-hour UOx was similar in the 4 patients who were able to provide samples (Table 3).

**Table 3:** Secondary efficacy endpoints.

	Lumasiran (*N* = 18)				
	Month 6	Month 12	Month 18	Month 24	Month 30
Change from baseline in spot UOx:Cr, mean (SEM)					
Absolute change from baseline, mmol/mmol^a^	−0.5 (0.1)	−0.5 (0.1)	−0.5 (0.1)	−0.5 (0.1)	−0.5 (0.1)
Percent change from baseline	−71.7 (3.4)	−71.9 (3.2)	−76.9 (3.9)	−75.4 (4.0)	−75.8 (4.5)
Patients with spot UOx:Cr, *n* (%)					
≤ULN^b^	1 (6)	2 (11)	3 (17)	3 (18)	7 (39)
≤1.5 × ULN^b^	9 (50)	10 (56)	11 (61)	7 (41)	13 (72)
Change from baseline corrected for BSA in 24-hour UOx, mean (SEM)^c^					
Absolute change from baseline mmol/24 h/1.73m^2^	−1.4 (0.1)	−1.2 (0.3)	−1.5 (0.1)	−1.6 (0.1)	−1.5 (0.4)
Percent change from baseline	−68.4 (5.6)	−63.2 (7.2)	−75.2 (4.3)	−72.9 (3.4)	−73.5 (8.8)
Absolute change from baseline in POx, mean (SEM)^d^					
In efficacy analysis set	−5.0 (1.3)	−7.3 (1.5)	−7.1 (1.6)	−6.3 (1.8)	−6.9 (1.6)
In POx analysis set^e^	−6.5 (1.6)	−9.5 (1.7)	−9.5 (1.8)	−9.0 (1.9)	−9.2 (1.8)
Percent change from baseline in POx, mean (SEM)^d^					
In efficacy analysis set	−32.1 (6.7)	−47.1 (4.6)	−42.6 (6.4)	−33.9 (10.7)	−42.5 (6.0)
In POx analysis set^e^	−37.4 (8.8)	−56.4 (3.8)	−55.6 (4.7)	−51.0 (7.0)	−53.0 (5.4)
Change from baseline in eGFR, mean (SEM), ml/min/1.73 m^2^f^^	−0.3 (3.8)	−1.5 (4.4)	−8.9 (3.6)	−3.2 (4.8)	−2.0 (4.7)

^a^ One mmol/mmol = 0.796 mg/mg; 1 mmol/mmol = 1,000 mmol/mol.

^b^ Age-dependent ULN (27).

^c^ In patients with valid 24-hour UOx measurements; *N* = 2 at Month 6, *N* = 4 at Month 12, *N* = 2 at Month 18, *N* = 3 at Month 24, *N* = 4 at Month 30.

^d^ ULN = 12.11 μmol/L for POx, as determined based on data from healthy adults (22).

^e^ In patients with baseline POx ≥1.5 × LLOQ [5.55 μmol/L (*N* = 13); values below LLOQ were assigned a value of 5.55 μmol/L].

^f^ eGFR (ml/min/1.73 m^2^) was calculated based on the Schwartz Bedside formula (29) for patients ≥12 months old; *N* = 16 at Month 6, *N* = 16 at Month 12, *N* = 16 at Month 18, *N* = 16 at Month 24, *N* = 15 at Month 30.

BSA, body surface area; eGFR estimated glomerular filtration rate; LLOQ lower limit of quantitation; POx, plasma oxalate; SEM, standard error of the mean; ULN, upper limit of normal; UOx, urinary oxalate; UOx:Cr urinary oxalate:creatinine ratio.

**Figure 2:**
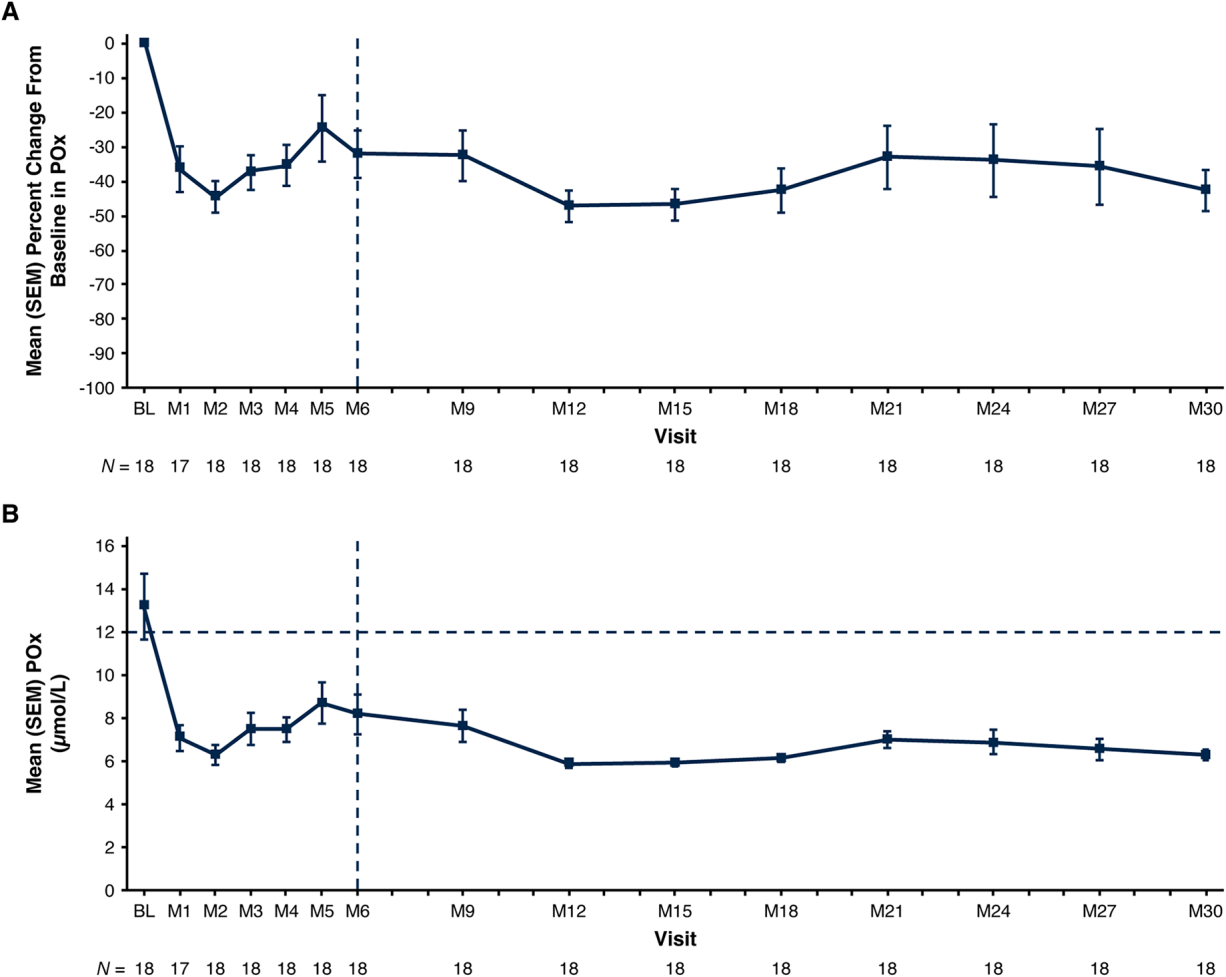
Mean (SEM) POx. **(A)** Percent change from baseline at each visit and **(B)** actual values at each visit. Baseline value represents the mean of all assessments collected prior to the first dose of lumasiran. The end of the primary analysis period is represented by the vertical dashed line. The ULN for POx, represented by the horizontal dashed line in panel B, is 12.11 μmol/L (determined based on data from 75 healthy adults) (22). The LLOQ is 5.55 µmol/L. Reductions in POx below the LLOQ were conservatively imputed as 5.55 µmol/L. BL, baseline; LLOQ, lower limit of quantitation; M, month; POx, plasma oxalate; SEM, standard error of the mean; ULN, upper limit of normal.

Mean POx decreased from 13.2 µmol/L at baseline to 6.3 µmol/L at Month 30 (ULN: 12.11 µmol/L); mean (SEM) percent change from baseline was −42.5% (6.0%) (Figure 2; Table 3). In patients with baseline POx ≥1.5 × LLOQ (*N* = 13), mean (SEM) POx decreased from 15.6 µmol/L at baseline to 6.4 µmol/L at Month 30; mean percent change from baseline was −53.0% (5.4%) (Table 3).

**Figure 3:**
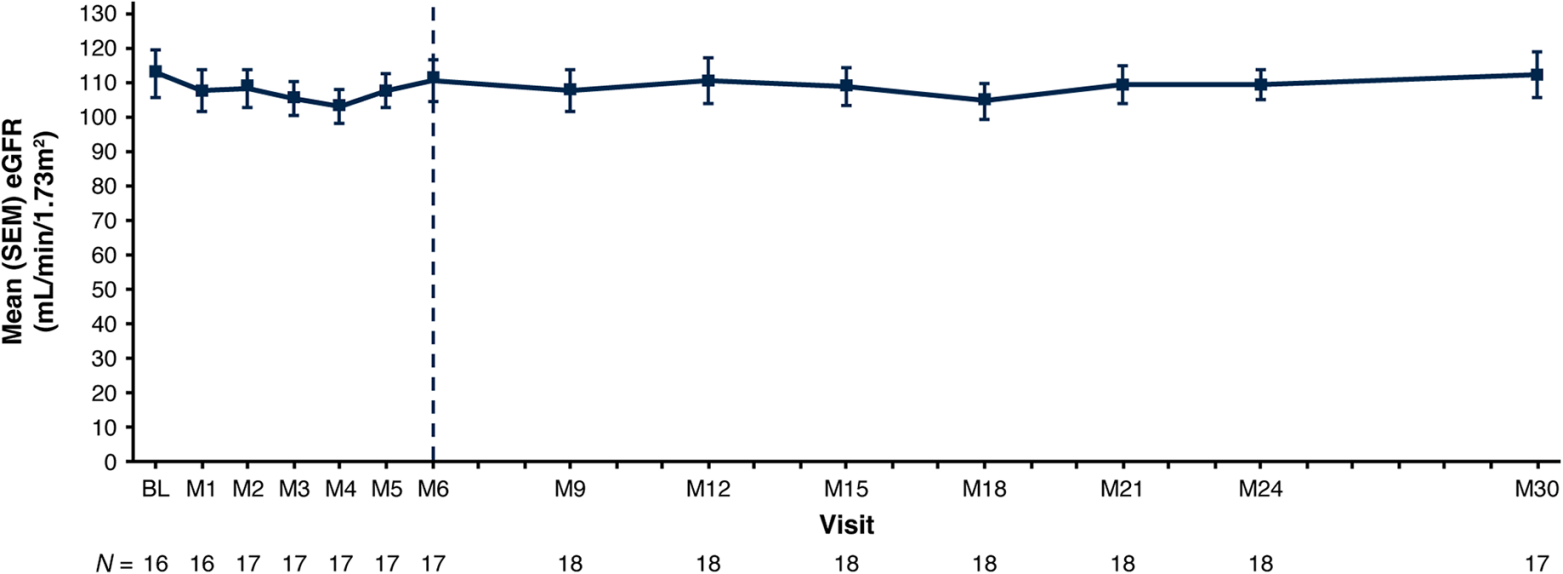
Mean (SEM) eGFR. Baseline is the last non-missing value collected prior to the first dose of lumasiran. The end of the primary analysis period is represented by the vertical dashed line. eGFR is calculated based on the Schwartz Bedside formula (29) in patients ≥12 months of age at the time of the assessment. BL, baseline; eGFR, estimated glomerular filtration rate; M, month; SEM, standard error of mean.

**Figure 4:**
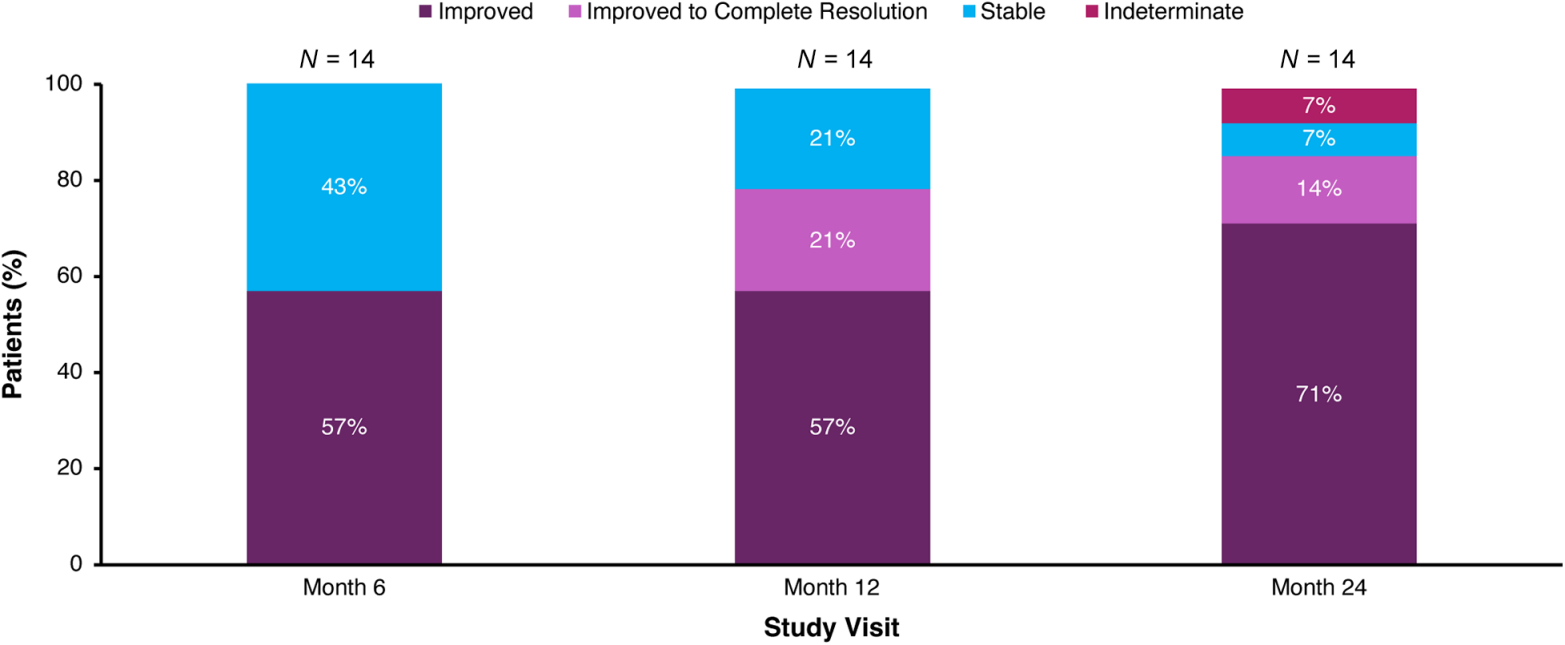
Change in medullary nephrocalcinosis grade in patients with nephrocalcinosis at baseline. Patients who had no nephrocalcinosis at baseline (*N* = 4) remained stable, with no nephrocalcinosis at Month 24; these patients are not depicted. Stable indicates grade same as baseline; improved indicates grade lower than baseline; and indeterminate indicates one side improved and the other side worsened. Renal ultrasound was not performed at Month 30.

**Figure 5:**
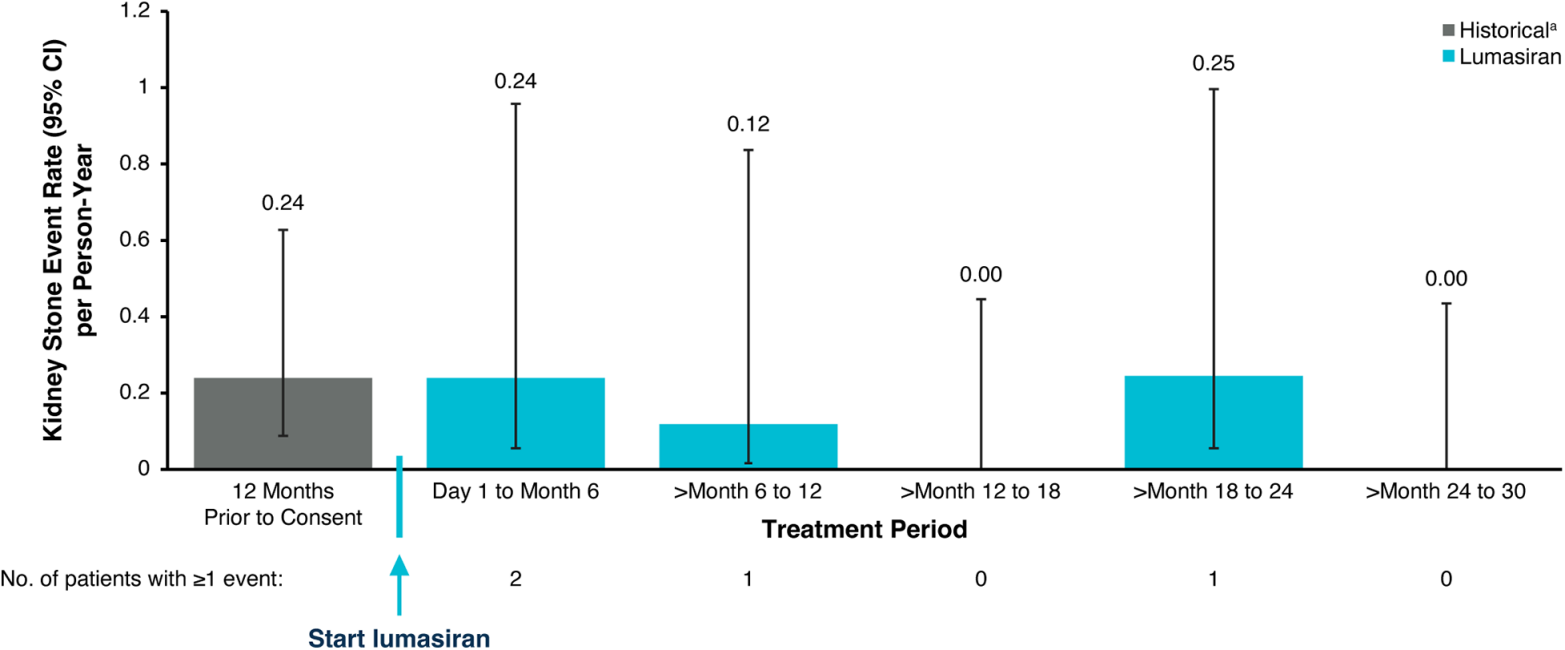
Kidney stone event rates. ^a^Historical patient-reported history of kidney stone events. An annualized rate was not calculated for patients <6 months old. CI, confidence interval.

eGFR remained stable with a mean (SEM) of 112.8 (6.9) ml/min/1.73 m^2^ at baseline and 112.5 (6.7) ml/min/1.73 m^2^ at Month 30 (Figure 3; Table 3). Nephrocalcinosis was present at baseline in 14 of 18 patients. Among the 14 patients with nephrocalcinosis at baseline, nephrocalcinosis grade improved at Month 24 in 12 (86%), was indeterminate in 1 (7%), and remained stable in 1 (7%) (Figure 4). Two of the 14 patients improved to complete resolution (Figure 4). The 4 patients who had no nephrocalcinosis at baseline remained stable, with no nephrocalcinosis at Month 24. Kidney stone event rates were ≤0.25 per person-year through Month 30 (Figure 5).

**Figure 6:**
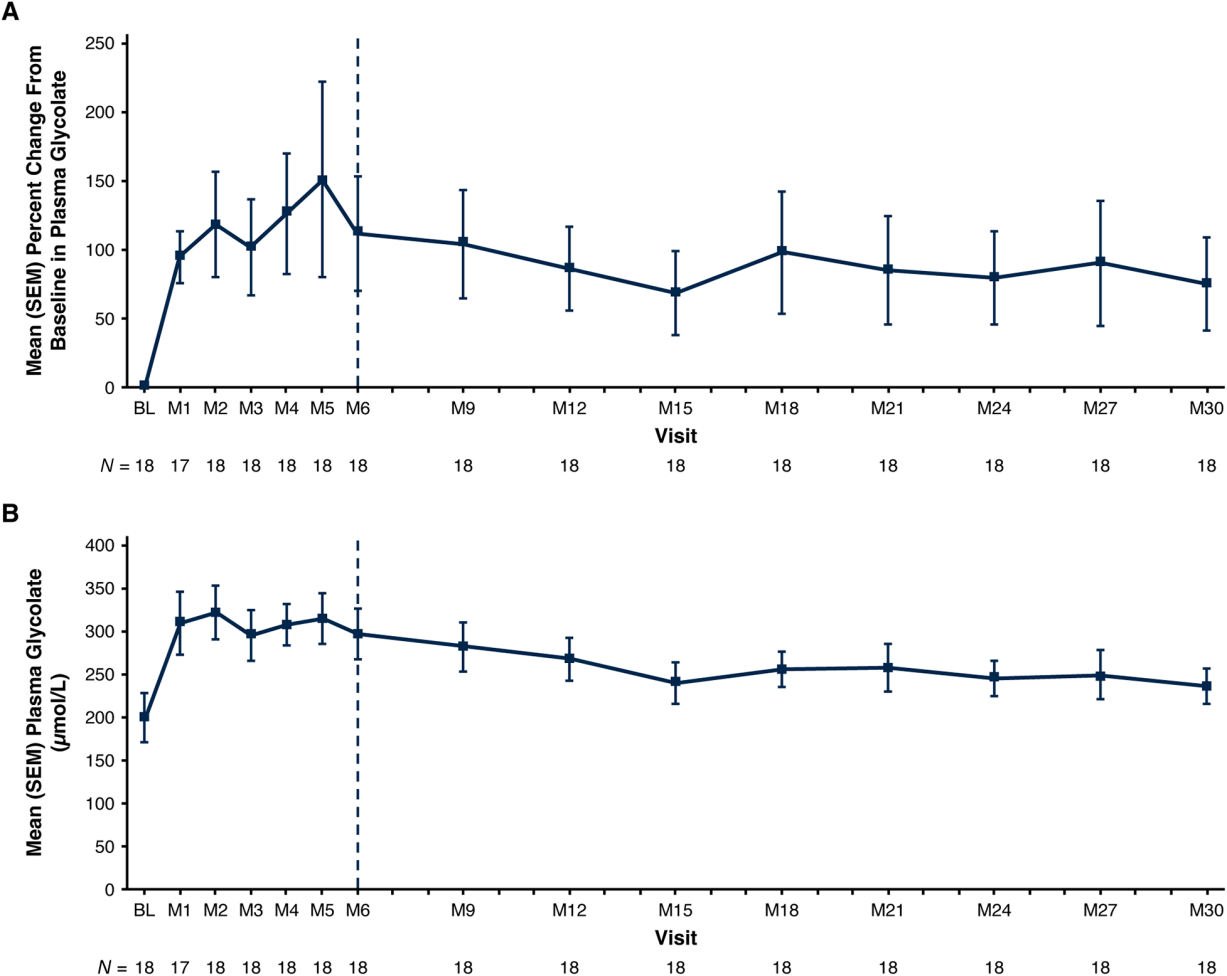
Mean (SEM) plasma glycolate. **(A)** Percent change from baseline at each visit and **(B)** actual values at each visit. Baseline value represents the mean of all assessments collected prior to the first dose of lumasiran. The end of the primary analysis period is represented by the vertical dashed line. BL, baseline; M, month; SEM, standard error of the mean.

Plasma glycolate initially increased, then plateaued, during the 6-month primary analysis period; thereafter, plasma glycolate declined slightly but remained elevated, as expected based on the mechanism of action of lumasiran (Figure 6).

Decisions regarding adjustments to hyperhydration and/or vitamin B6 regimens after Month 6 were left to the discretion of study investigators. Three of 13 patients on hyperhydration at baseline decreased it during the extension period; no patients started hyperhydration during the study. After Month 6, 5 of 11 patients taking vitamin B6 at baseline stopped vitamin B6, 2 reduced their dose without stopping, and no patients started vitamin B6. There was no meaningful change in UOx:Cr ratios in patients who decreased or stopped hyperhydration or vitamin B6.

### 3.3 Safety

As of the cutoff date (April 29, 2022), median (range) exposure to lumasiran was 32.6 (27.5‒35.3) months. Five (28%) patients had AEs deemed by the investigator to be related to lumasiran (Table 4). The most common lumasiran-related AEs were mild, transient injection site reactions [3 patients (17%)]; symptoms included erythema, discoloration, and pain at the injection site. One patient had a serious AE of viral infection (moderate in severity and considered unrelated to lumasiran), as reported previously (23).

**Table 4:** Safety profile of lumasiran.

	All treated (*N* = 18)
AEs	18 (100)
Treatment-related AEs^a^	5 (28)
AEs leading to treatment discontinuation	0
AEs leading to study withdrawal	0
Serious AEs	1 (6)^b^
Severe AEs	0
Death	0

^a^ Treatment-related AEs included injection site reactions, transient blood bilirubin increase, and headache.

^b^ One patient had a serious AE of viral infection (moderate in severity; considered unrelated to lumasiran by the investigator) during the 6-month primary analysis period, which was reported previously (22).

AE, adverse event.

There were no clinically relevant changes related to lumasiran in laboratory measures, vital signs, or electrocardiograms. One patient had an AE of blood bicarbonate decreased that was deemed by the investigator to be unrelated to lumasiran. At baseline, the patient had an eGFR of 134 ml/min/1.73 m^2^ and a bicarbonate value of 19 mmol/L, and was on a stable dose of oral sodium bicarbonate for PH1. The AE of blood bicarbonate decreased was entered due to a bicarbonate of 18 mmol/L at Month 12; the dose of oral sodium bicarbonate was not changed. The AE was considered resolved after the bicarbonate value at Month 15 was 21 mmol/L. Plasma glycolate remained stably elevated. The patient received no treatment for the AE and remained on lumasiran.

Transient, low-titer (1:50) anti-drug antibodies were observed in 3 (17%) patients, with no observed impact on safety or efficacy. None of the patients tested positive for anti-drug antibodies at baseline.

## 4 Discussion

Lumasiran, the first approved treatment for PH1 (17, 18) and the first RNAi therapeutic to be studied and approved in infants and young children (22), is a disease-modifying therapy that addresses the source of hepatic oxalate overproduction in PH1 by substrate reduction leading to decreased hepatic oxalate synthesis (19). Long-term treatment with lumasiran was associated with sustained lowering of UOx excretion, stable renal function (eGFR), and improvements in medullary nephrocalcinosis in patients with PH1 who were <6 years of age and had an eGFR >45 ml/min/1.73 m^2^ at baseline. Kidney stone event rates remained low. Plasma glycolate levels remained elevated, consistent with reduced hepatic glycolate oxidase activity mediated by lumasiran; there are no known adverse consequences of elevated glycolate concentrations in blood (22, 23, 32).

Hyperhydration, or large daily fluid intake (proportionate to body size in children), may attenuate the effects of hyperoxaluria (9). However, hyperhydration requires a gastrostomy tube or nasogastric tube in some young children and negatively impacts quality of life; hence, adherence may be poor (9, 10, 33). In this study, hyperhydration status was recorded for all patients; 3 patients on hyperhydration decreased hyperhydration during the extension period, and none started it. Reducing the need for hyperhydration is likely to increase quality of life in patients with PH1 (10).

Vitamin B6 is recommended for patients with PH1 who have a vitamin B6–responsive genotype; it has been associated with a mean decrease in UOx of approximately 26% (34). In this study, 5 of 11 patients taking vitamin B6 at baseline stopped vitamin B6, 2 reduced their dose, and none started vitamin B6. Of the 5 patients who stopped taking vitamin B6, 3 had a pyridoxine-responsive genotype and 2 did not. This apparent reduction in the need for vitamin B6 with maintenance of UOx:Cr suppression strengthens the evidence for the efficacy of lumasiran.

Lumasiran demonstrated an acceptable safety profile; injection site reactions were the most commonly reported AE. There was only one serious AE (a viral infection) reported as of the Month 30 data cutoff, and it was not considered related to lumasiran. These findings suggest that RNAi therapy is safe for use in infants and small children. This is corroborated by recent case reports of lumasiran use in infants (35, 36) and young children (37).

Transient, low-titer (1:50) anti-drug antibodies were observed in 3 patients during the study. Similar findings of low-titer anti-drug antibodies in a minority of patients have been reported in other clinical studies of lumasiran, and, when assessed, no effect of anti-drug antibodies on lumasiran pharmacokinetics has been noted (22, 38–40). There was no observed impact of the anti-drug antibodies on efficacy or safety in this or other studies (22, 38, 40).

## 5 Conclusions

In infants and young children with PH1, lumasiran treatment resulted in reductions in UOx and POx that were maintained through Month 30. The safety profile of lumasiran was acceptable. Clinical assessments of kidney health were encouraging, including stable kidney function through Month 30 and improvement in nephrocalcinosis through Month 24. Kidney stone event rates were low through Month 30. The most common lumasiran-related AEs were mild, transient injection site reactions.

## Data Availability

Access to anonymized individual participant data that support these results is made available 12 months after study completion and not less than 12 months after the product and indication have been approved in the US and/or the EU. Requests for access to data can be submitted via the website http://www.vivli.org.
